# Drought-induced alterations in photosynthetic, ultrastructural and biochemical traits of contrasting sugarcane genotypes

**DOI:** 10.1371/journal.pone.0235845

**Published:** 2020-07-08

**Authors:** Yue-Bin Zhang, Shao-Lin Yang, Jing-Mei Dao, Jun Deng, Ahmad Naeem Shahzad, Xian Fan, Ru-Dan Li, Yi-Ji Quan, Syed Asad Hussain Bukhari, Zhao-Hai Zeng

**Affiliations:** 1 College of Agronomy and Biotechnology, China Agricultural University, Beijing, China; 2 Sugarcane Research Institute of Yunnan Academy of Agricultural Sciences, Kaiyuan, Yunnan, China; 3 Department of Agronomy, Bahauddin Zakariya University, Multan, Pakistan; Harran University, TURKEY

## Abstract

Drought is an important factor which limits growth of sugarcane. To elucidate the physiological and biochemical mechanisms of tolerance, a pot experiment was conducted at Sugarcane Research Institute, Kaiyuan, China. Two genotypes (Yuetang 93-159-sensitive and Yunzhe 05-51-tolerant), were subjected to three treatments; 70±5% (control), 50±5% (moderate drought) and 30±5% (severe drought) of soil field capacity. The results demonstrated that drought induced considerable decline in morpho-physiological, biochemical and anatomical parameters of both genotypes, with more pronounced detrimental effects on Yuetang 93–159 than on Yunzhe 05–51. Yunzhe 05–51 exhibited more tolerance by showing higher dry biomass, photosynthesis and antioxidant enzyme activities. Compared with Yuetang 93–159, Yunzhe 05–51 exhibited higher soluble sugar, soluble protein and proline contents under stress. Yunzhe 05–51 illustrated comparatively well-composed chloroplast structure under drought stress. It is concluded that the tolerance of Yunzhe 05–51 was attributed to improved antioxidant activities, osmolyte accumulation and enhanced photosynthesis. These findings may provide valuable information for future studies on molecular mechanism of tolerance.

## Introduction

Sugarcane (*Saccharum sp*.) possesses great economic significance in the world due to its large-scale applicability in sugar industry and manufacturing of ethanol an environment friendly renewable source of energy [1]. Sugarcane contributes about 90% to national sugar production in China. Yunnan is one of the main sugarcane producing regions in China, which is characterized with uneven and sloping uplands with shallow plough layer and no irrigation system [2]. In China, water deficit is the main hindrance in sugarcane production because of its large-scale cultivation on upland regions, where unavailability of irrigation water is a big challenge. Drought spells, especially during spring and autumn, severely impede sugarcane productivity, or even lead to no yield at all under extreme circumstances [3, 4].

Beyond certain limits, water deficit causes a significant disruption in physiological performance of plants [5, 6]. Photosynthetic process is extremely prone to scarcity of moisture [7, 8] due to reduction in exchange rate of carbon dioxide and damage to chloroplast structure under severe stress level [3, 9]. Most of the water absorbed by plants is transpired by leaves and being the site for photosynthesis, their physiological activities are highly vulnerable to water deficit, notably stomatal conductance and transpiration that acts decisively on photosynthesis [10]. Zhang *et al*. [3] reported a clear deterioration in the chloroplast structure of sugarcane leaves under water deficit conditions. Drought-induced alterations in ultrastructural morphology of cotton leaves have also been reported by previous researchers [11]. However, the severity of detrimental outcomes of stress on ultrastructural morphology of plants is variable that depends upon the tolerance level of different genotypes [12]. The anatomical structure of two sugarcane genotypes, with varying degree of tolerance, revealed a conspicuous difference in response to drought stress. The drought-induced distortion in chloroplast structure of sensitive genotype (YL6) was severer than that of tolerant (F172) one [3]. Besides chloroplast damage, photosynthetic efficiency of plants is also impaired by stomatal closure [13] and disrupted biochemical reactions under limited moisture [14]. Nonetheless, the deleterious effects of environmental stresses on photosynthetic efficiency of plants are variable in different genotypes within the same species. Rodrigues *et al*. [15] reported a considerable variation in photosynthetic efficiency of different sugarcane genotypes under water deficit conditions. In another study, Sales *et al*. [16] reported a differential response of sugarcane genotypes to drought stress in terms of photosynthesis and antioxidant enzyme activities. The magnitude of impairment in photosynthetic efficiency of sensitive genotypes is more pronounced than that of tolerant ones [17, 18]. Plants have evolved various morphological, physiological and biochemical mechanisms to cope with the adverse environmental conditions [13]. Water deficit also induces oxidative stress in plants which is ameliorated by internal enzymatic and non-enzymatic antioxidants by scavenging the reactive oxygen species [19, 20]. Drought tolerant genotypes demonstrate comparatively higher antioxidant activities than the susceptible ones [18, 21]. Environmental stresses trigger several physiological and biochemical responses in plants as a strategy to withstand adverse circumstances [22]. Regarding biochemical responses to moisture deficit, plants accumulate solutes like proline, sugars, along with activation of antioxidant defense system [16, 23, 24].

The objective of the current investigation was to test the hypothesis that genotypic difference in drought tolerance of sugarcane was associated with effective antioxidant defense system, which might protect the photosynthetic machinery of plants under drought conditions.

## Materials and methods

### Plant materials and treatments

The experiment was carried out using a completely randomized experimental design, 2 × 3 factorial arrangement (two genotypes × three soil field capacity: control, moderate drought and severe drought). The sugarcane cultivars Yuetang 93–159 (drought sensitive) and Yunzhe 05–51 (drought tolerant) were grown in pots in wire house under natural light conditions at Yunnan Sugarcane Research Institute, Kaiyuan (23°42ʹN, 103°15ʹE, Elevation 1050 m), China during 2019. Three single budded sets were sown in every pot having 20 kg soil. The pots were 29 cm deep with 35cm upper and 28 cm lower diameter, having 5 evenly spaced holes in the bottom. The soil was fertilized at the rate of 100 g NPK (15:15:15) per pot. The experiment was replicated thrice. Initially, the plants were grown normally under well-watered conditions. At elongation stage, the pots with healthy and morphologically alike plants were chosen for the experiment. The plants were subjected to stress treatments at day 100 from germination. The plants were subjected to varying levels of soil moisture on field capacity (F.C) basis: (1) control, 70±5% of the soil F.C; (2) moderate drought, 50±5% of the soil F.C; (3) severe drought, 30±5% of soil FC. The pots were weighed every day and replenished with required quantity of water to maintain the above-mentioned moisture level for 10 days. Soil physico-chemical characteristics were as follows:

16.8% Sand, 21% silt, 62.2% clay, 7.2 pH, 0.104% N, 0.124% total P and 2.65% K, measured using routine analytical methods [25].

### Morphological parameters

After recording data on number of leaves, plant height and leaf area plants were harvested to measure the shoot fresh weight. The green leaf area of plants was determined as follows:

leaf area = (length × width) × 0.71 [26, 27].

The plant shoots were placed in oven for 72 hours at 70°C to determine their dried biomass.

### Gas exchange attributes, chlorophyll content and relative water content

A portable photosynthesis system Li-6400 (LI-COR Biosciences, Lincoln, NE, USA) was employed to measure the net photosynthesis (Pn), intercellular CO_2_ concentration (Ci), stomatal conductance (Gs) and transpiration rate (Tr). All the measurements were taken on the same clear sunny day between 9.00 am to 12.00 a.m., with 25°C air temperature, 80–90% relative humidity, 400 μmol mol^-1^ CO_2_ concentration and 1000 μmol m^-2^ s^-1^ Photosynthetic photon flux density. Chlorophyll content was recorded on the middle part of the youngest leaves with visible dewlap using chlorophyll meter SPAD-502 Plus (Konica Minolta Optics, INC, Japan). The detached leaves were weighed immediately, followed by soaking in water at room temperature for 8 hours to record their turgid weight [28]. The same leaves were subjected to 70°C for 3 days to determine their dry weight. The relative water content (RWC) of leaves was derived using the following formula given by Barrs and Weatherley [28]:

RWC=[(FW−DW)/(TW−DW)]×100

where, FW, DW and TW represent the fresh weight, dry weight and turgid weight of leaves, respectively.

### Enzyme activity assay

The samples collected from uppermost leaves with visible dewlap were immediately immersed in liquid N_2_, followed by storage at -80°C for subsequent biochemical assay. For antioxidant enzymes activity assay, the samples (0.3 g) were macerated in 50 mM phosphate buffer solution (PBS, 50mM, pH 7.8). The extract of samples was subjected to centrifuge at 10,000×g for 15 minutes and the supernatant was shifted to new tubes to determine the activities of antioxidant enzymes. Superoxide dismutase (SOD, EC 1.15.1.1) activity was determined according to the method suggested by Dhindsa et al. [29] by detecting the inhibition of NBT (nitroblue tetrazolium) reduction. The reaction mixture contained 75 μM NBT, 130 mM methionine, 20 μM riboflavin, 100 μM EDTA and enzyme extract. The mixture was exposed to fluorescent lamps for 20 minutes before measuring the absorbance at 560 nm. For peroxidase (POD, EC 1.11.1.7) activity determination, the reaction mixture was consisted of 50 mM PBS (pH 7.8), 1.5% guaiacol, 300 mM H_2_O_2_ and enzymes extract. The spectrophotometer was used to detect the absorbance at 470 nm [30]. The activity of catalase (CAT, EC 1.11.1.6) was quantified as described by Aebi [31]. Absorbance of the reaction mixture (50 mM PBS at pH 7.8, enzyme extract and 300 mM H_2_O_2_) was detected at 240 nm optical density as an outcome of H_2_O_2_ disappearance. The ascorbate peroxidase (APX, EC 1.11.1.11) activity was detected by observing the ascorbic acid-induced reduction of H_2_O_2_ at 265 nm. The reaction mixture was prepared with 50 mM PBS, enzyme extract, 7.5 mM ASA and 300 mM H_2_O_2_ [32].

### Relative electrolyte leakage, lipid peroxidation and reactive oxygen species

The small leaf discs (approximately 0.5 mm diameter) were collected in tubes having 8 ml distilled water. The initial electrical conductivity (EC1) of the medium was recorded after 2 hours incubation at 32°C. The samples were then autoclaved for 20 minutes at 121°C. After cooling the samples to room temperature, the electrical conductivity (EC2) was again noted using the conductivity meter (DDSJ-308A, Westtune, China). The electrolyte leakage (EL) was computed by employing the equation {EL = (EC1/EC2) ×100} proposed by Dionisio-Sese and Tobita (1998). Lipid peroxidation was estimated by analyzing the malondialdehyde (MDA) content using the protocol given by Dhindsa *et al*. (1981). Leaf tissues (0.25 g) were macerated in 0.1% trichloro acetic acid (TCA), followed by centrifugation for 5 minutes at 10000g. The supernatant (1 mL) was mixed with 4 mL of 0.5% TBA, prepared in 20% solution of TCA. After incubating at 95°C for 30 minutes, the mixture was placed in ice bath. The mixture was then again subjected to centrifugation for 10 minutes at 10,000xg. The supernatant was run on spectrophotometer and the values of non-specific absorbance (600 nm) were deducted from those recorded at 532 nm. The MDA contents were computed using the extinction coefficient (155 mM^−1^ cm^−1^). For hydrogen peroxide (H_2_O_2_) contents, the leaf samples were crushed in 50 mM PBS (6.5 pH), followed by centrifugation for 25 minutes at 6000xg. The supernatant was mixed with titanium sulphate (0.1% in 20% H_2_SO_4_) solution and centrifuged for 15 minutes (6000g). The supernatant was analyzed on spectrophotometer at 410 nm optical density and H_2_O_2_ contents were quantified by applying extinction coefficient (0.28 μmol^−1^ cm^−1^) [33]. Superoxide radical (O^-^_2_) was analyzed following Gill *et al*. [34]. Sixty-five mM potassium phosphate buffer (pH 7.8) was used to homogenize leaf samples (0.5 g). The homogenate was subjected to centrifuge for 10 minutes (5000g, 4°C). Hydroxylamine hydrochloride (10 mM) and potassium phosphate buffer were mixed with the supernatant and kept for 24 hours at 25°C. After incubation, sulphanilamide (17 mM) and a-naphthylamine (7 mM) were mixed with the solution and kept at 25°C for another 20 minutes. After incubation, n-butanol was mixed with the solution and centrifuged for 5 minutes at 1500g. The absorbance of extract was detected spectrophotometrically at 530 nm. A standard curve was used to determine the O^-^_2_ contents.

### Proline, soluble sugars and soluble proteins

Sulphosalycylic acid (3% w/v) was used for proline extraction from sugarcane leaf tissues (0.5 g). Proline content was determined using the ninhydrin reagent following the procedure given by Bates *et al*. [35]. The absorbance of fraction with toluene, released from the liquid phase, was noted at 520 nm. A calibration curve was used to determine the proline contents. Anthrone method was utilized to analyze the soluble sugar contents in leaf tissues [36]. Leaf samples (0.5 g) were macerated and mixed with 5 mL of 80% ethanol. After centrifugation at 9000g for 15 minutes, the extract was shifted to new tubes and mixed with 12.5 ml of 80% ethanol. The solution (1 ml) was added to 1 ml anthrone (0.2%). The solution was heated at 100°C for 20 minutes and placed in ice for 5 minutes thereafter to stop the reaction. The absorbance of the solution was recorded at 620 nm and soluble sugar contents were quantified using a standard curve. The leaf soluble protein contents were assayed using the protocol introduced by Bradford [37]. The samples (0.5g) were crushed in 0.1M PBS (pH 7.0). The homogenate was subjected to centrifuge for 20 minutes at 1000 rmp. The supernatant was mixed with dye mixture and kept for 15 minutes. The absorbance was detected spectrophotometrically at 595 nm. Dye mixture was composed of 100 mg Coomassie brilliant blue (G 250), dissolved in 50 mL ethanol (95%) and 100 mL ortho phosphoric acid. Distilled water was added to make the volume 200 ml. 1 ml dye mixture was added in 4 ml distilled water to analyze the samples.

### Transmission electron microscopy

Leaf fragments were excised from top leaves with visible dewlap and then fixed in 2.5% glutaraldehyde (v/v), prepared with 0.1M PBS (pH 7.0), for 24 hours. After giving 3 washings with the same PBS, the leaf fragments were fixed in 1% osmium tetroxide, followed by 3 times rinsing with the same PBS. Then dehydration of the samples was done using a graded series of ethyl alcohol (50, 70, 80, 90, 95 and 100%) for 15 minutes every time. After overnight embedding in Spurr’s resin, the samples were heated at 70°C for 16 h. Ultra-thin sections (80 nm) of samples were cut before mounting on copper grids to visualize under transmission electron microscope (JEOL TEM-1230EX, Japan) at an accelerating voltage of 60.0 kV [38].

### Statistical analysis

Data were subjected to Fisher’s analysis of variance using Statistix 8.1 Multiple comparisons to separate treatment means were performed using the least significant difference test with P≤5%.

## Results

### Growth attributes

**Fig 1:**
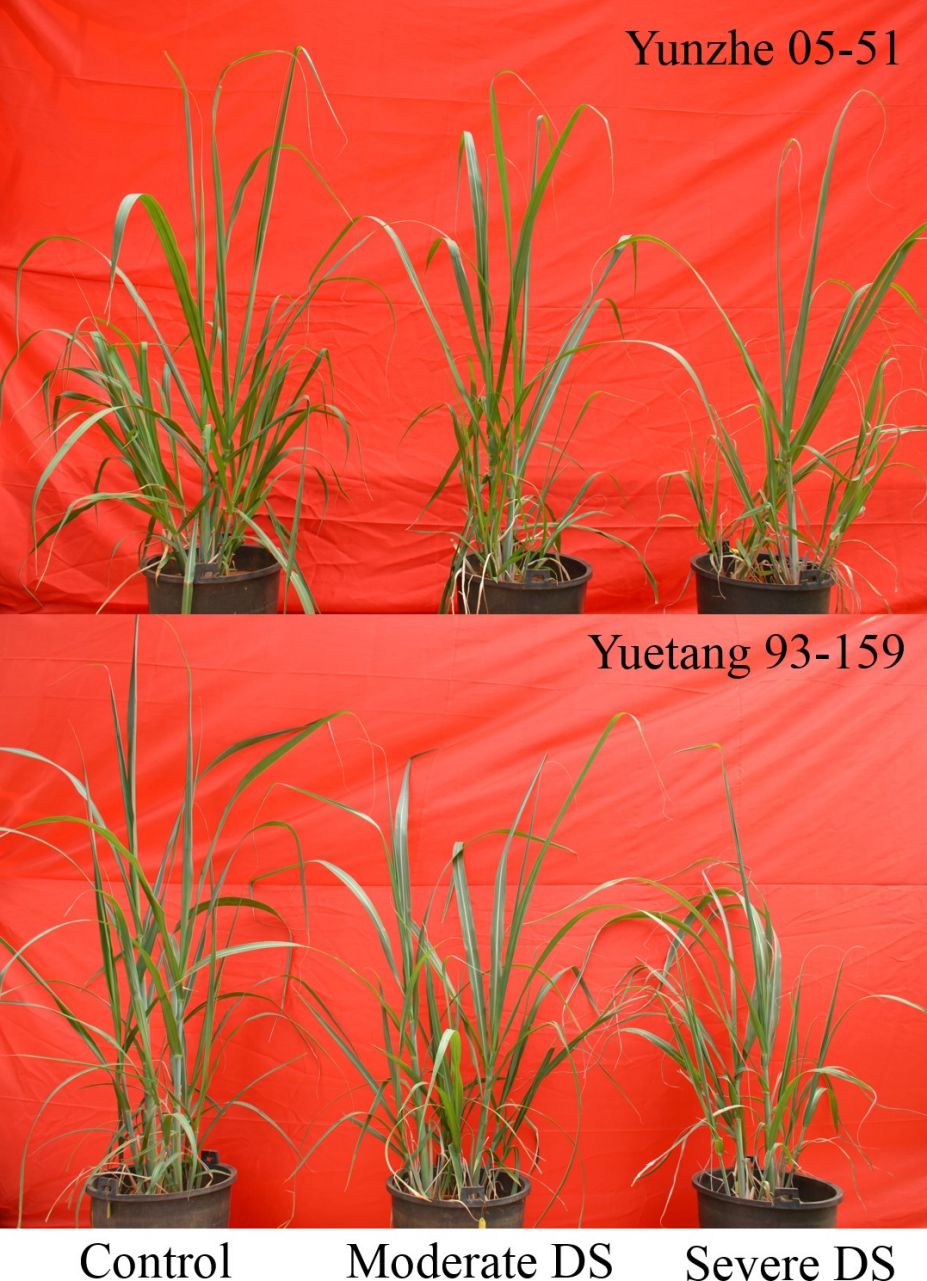
Phenotypes of 2 sugarcane genotypes under control (70±5%), moderate drought stress (50±5%) and severe drought stress (30±5%) of soil field capacity.

Increasing drought stress clearly reduced the height of sugarcane plants of both varieties. Under severe drought conditions (30±5% FC), the reduction in plant height was 20.63% and 15.14% in Yuetang 93–159 and Yunzhe 05–51, respectively, in relation to their respective control plants receiving sufficient irrigation. Nonetheless, the difference between two varieties for plant height was statistically non-significant (Table 1 and Fig 1). Water deficit considerably declined the number of leaves of Yuetang 93–159 in relation to the control plants, but showing a statistically non-significant difference between moderate and severe stress treatments. Yunzhe 05–51 showed statistically non-significant difference among the treatments. Green leaf area of plants depicted a clear difference between treatments in both cultivars facing water scarcity, in contrast to their respective well-watered plants. But the effect of moderate and severe drought stress was statistically at par with each other. However, no statistical difference was observed between genotypes.

**Table 1:** Drought-induced differences in morphological parameters of 2 sugarcane genotypes.

Treatment	Plant height (cm)		No. of leaves plant^-1^	Leaf area plant^-1^ (cm^2^)	Shoot fresh weight (g plant^-1^)	Shoot dry weight (g plant^-1^)
**Yuetang 93–159**						
Control	173±17 aA	14.8±1.52 aA		4251±826 aA	350±25 aA	73±5.8 aA
Moderate drought	150±9 bA	9.3±1.04 bA		1855±372 bA	183±15 bB	43±4.7 bA
Severe drought	137±5 bA	9.0±1.32 bA		1648±136 bA	167±13 bB	37±4.6 bB
**Yunzhe 05–51**						
Control	168±12 aA	12.7±1.75 aA		3405±399 aA	357±19 aA	70±6.3 aA
Moderate drought	155±4 abA	10.8±1.04 aA		2292±777 bA	231±18 bA	51±5.9 bA
Severe drought	143±7 bA	10.2±0.76 aA		1817±55 bA	213±11 bA	48±4.6 bA
LSD						
Drought	13.9**	1.7***		710***	21.9***	6.7***
Variety	Ns	ns		ns	17.9**	5.4*
Interaction	Ns	ns		ns	Ns	ns

ns: No significant effects.

*Significant effect at P < 0.05 level.

**Significant effect at P < 0.01 level.

***Significant effect at P < 0.001 level.

Values are means of three replicates ± SD. Treatment means followed by different letters for genotypes within each water condition (uppercase) as water condition within genotypes (lowercase) differ statistically at P≤5%. Control, moderate drought and severe drought represent 70±5%, 50±5% and 30±5% of soil field capacity, respectively.

Both sugarcane genotypes exhibited a decrease in shoot fresh weight of plants, with more noticeable effect on Yuetang 93–159. At moderate drought stress treatment, 47.6% and 35.3% reductions were noted in Yuetang 93–159 and Yunzhe 05–51, respectively. While at severe stress level, shoot fresh weight declined to 52.14% and 40.39% in Yuetang 93–159 and Yunzhe 05–51 respectively, as compared with their respective control plants. Both water deficit treatments prominently reduced the dry biomass of plant shoots. But the genotypes responded differentially to the applied drought stress. Facing severe water deficit, sensitive genotype exhibited a colossal reduction in dry biomass as compared with tolerant one. Yuetang 93–159 showed 41.4% and 50.09% while Yunzhe 05–51 depicted 27.78% and 30.84% curtailment in shoot dry weight at moderate and acute stress conditions, respectively.

### Gas exchange parameters, SPAD and water use efficiency

**Fig 2:**
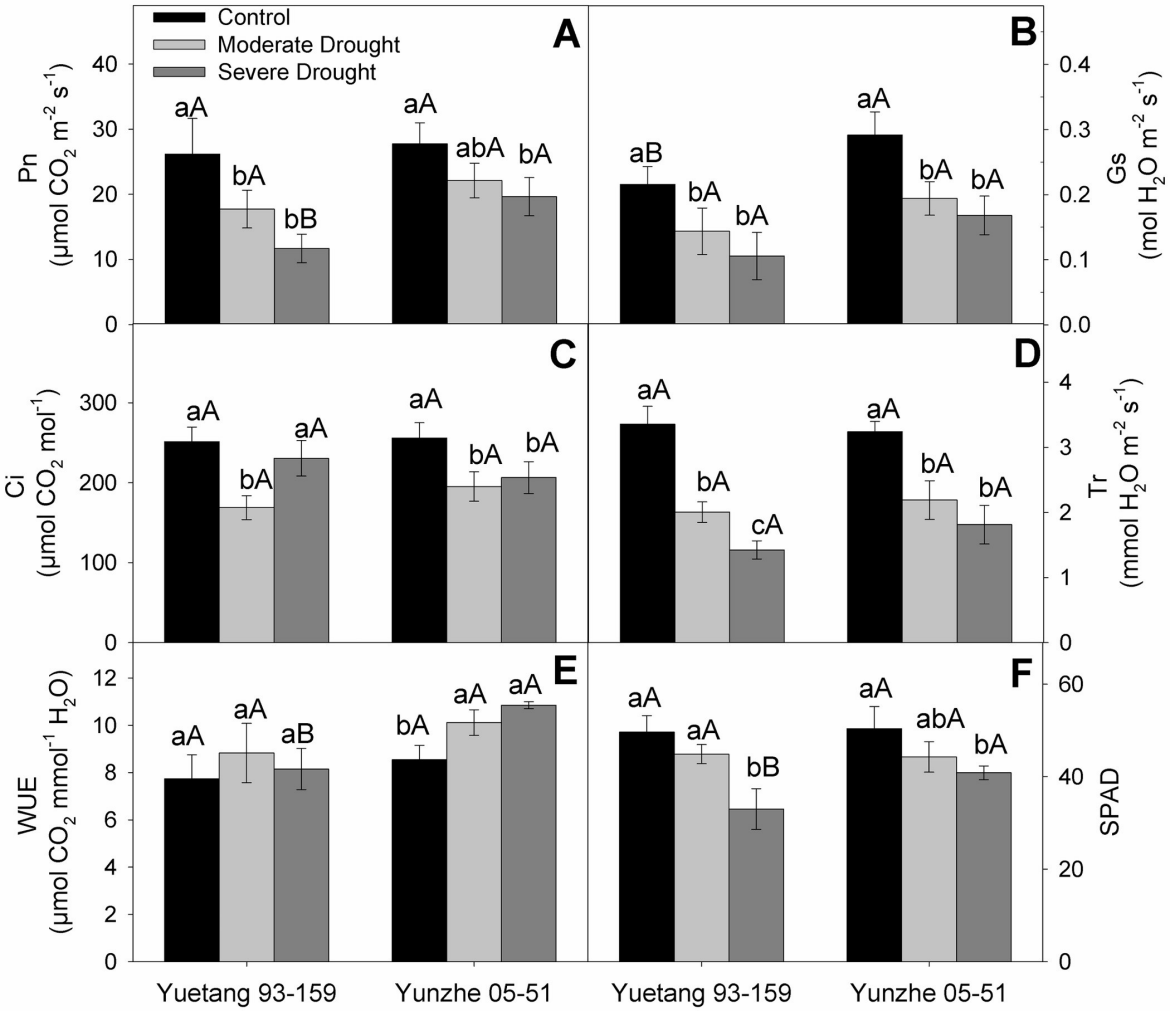
Photosynthetic attributes of two sugarcane genotypes in response to drought stress. (A) Net photosynthesis (*P*n), (B) Stomatal conductance (*G*s), (C) Intercellular CO_2_ concentration (*C*i), (D) Transpiration rate (*Tr*), (E) Water use efficiency (WUE) and (F) Chlorophyll content (SPAD). Columns represent the mean values of three replicates ± SD. Bars followed by different letters for genotypes within each water condition (uppercase) as water condition within genotypes (lowercase) differ statistically at P≤5%. Control, moderate drought and severe drought represent 70±5%, 50±5% and 30±5% of soil field capacity, respectively.

Both varieties faced a significant decline in net photosynthetic rate (*P*n) under water deficit conditions. At moderate and severe drought stress, *P*n reduced significantly in Yuetang 93–159. Conversely, tolerant genotype showed decline in *P*n only at severe water deficit with respect to control. However, at severe stress level, the tolerant genotype (Yunzhe 05–51) revealed considerably higher photosynthetic rate as compared to the sensitive one (Yuetang 05–51) (Fig 2A). Drought-stressed plants of both sugarcane genotypes showed a significant reduction in stomatal conductance (*G*s) in relation to their respective control treatments. However, the difference between moderate and severe stress levels was statistically negligible. Both varieties responded similarly to water deficit treatments in terms of *G*s (Fig 2B). The results revealed a marked impact of water scarcity on internal CO_2_ concentration (*C*i) of both varieties. Moderate moisture deficiency reduced the *C*i in both varieties with respect to control. At severe stress level, *C*i increased in both genotypes as compared with the moderate stress level. (Fig 2C). A considerable variation was observed between the drought sensitive (Yuetang 93–159) and tolerant (Yunzhe 05–51) sugarcane genotypes in terms of transpiration rate (*T*r) in response to moisture deficit conditions. Both stress levels (moderate and severe drought) significantly reduced the transpiration rate. But the outcomes of stress proved to be more detrimental to Yuetang 93–159 (40% and 58% decrease by moderate and severe drought, respectively) as compared to the Yunzhe 05–51 (32% and 44% decrease by moderate and severe drought, respectively) (Fig 2D). Data on water use efficiency (WUE) revealed clear differences between two sugarcane genotypes under stress. The tolerant genotype (Yunzhe 05–51) showed significantly enhanced WUE at both stress levels in relation to the respective control treatment. However, the difference between moderate and severe water deficit was not noteworthy statistically. On the other hand, no improvement was observed in WUE of drought-sensitive genotype (Yuetang 93–159) at both stress treatments, as compared with the fully watered plants (Fig 2E). Both cultivars exhibited statistically non-significant reduction in SPAD value at moderate water deficit stress, as compared with their respective control treatments. Severe drought stress further reduced the SPAD value in both genotypes. However, Yunzhe 05–51 showed considerably higher SPAD value than Yuetang 93–159 at severe stress level (Fig 2F).

### Electrolyte leakage, relative water content, reactive oxygen species and MDA

**Fig 3:**
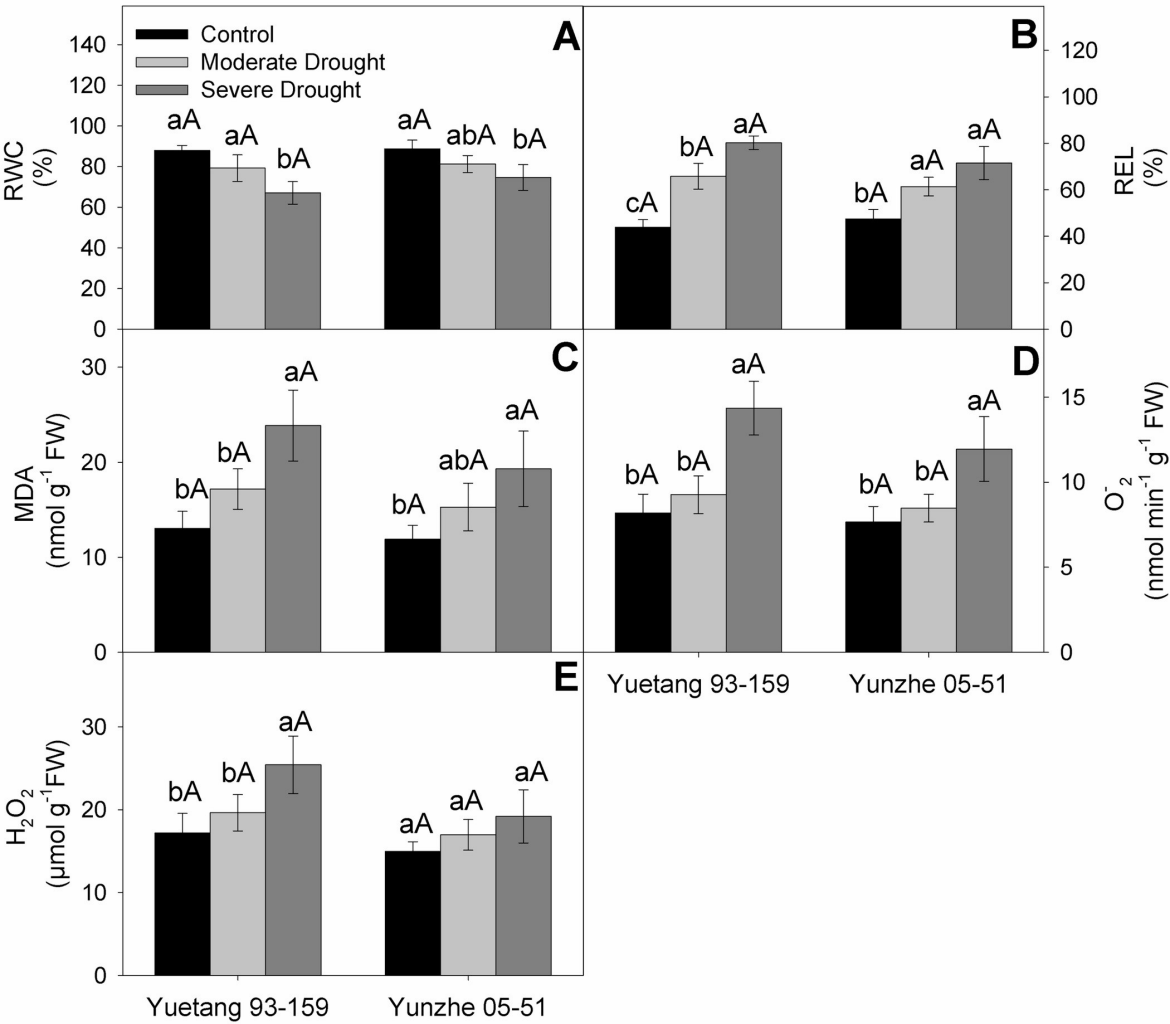
Physiological and ROS attributes of two sugarcane genotypes in response to drought stress. (A) Relative water content (RWC), (B) Relative electrolyte leakage (REL), (C) Lipid peroxidation (MDA), (D) Superoxide (O^-^_2_) and (E) Hydrogen peroxide (H_2_O_2_). Columns represent the means values of three replicates ± SD. Bars followed by different letters for genotypes within each water condition (uppercase) as water condition within genotypes (lowercase) differ statistically at P≤5%. Control, moderate drought and severe drought represent 70±5%, 50±5% and 30±5% of soil field capacity, respectively.

Both genotypes depicted a reduction in leaf relative water content (RWC) under reduced moisture availability. The reduction in RWC of both genotypes was not statistically noticeable at moderate stress treatment, compared with the control. However, a clear reduction in RWC was noted in both varieties at severe water deficit treatment. However, no statistical difference was observed in RWC of two varieties under various treatments (Fig 3A). The results revealed that increasing drought stress escalated the relative electrolyte leakage (REL) in both sugarcane cultivars. Comparing with the normally irrigated ones, the stressed plants of Yuetang 93–159 showed a significant increase in leaf REL at moderate stress level which was further exacerbated at severe water deficit treatment. Nonetheless, drought tolerant genotype (Yunzhe 05–51) depicted a rise in leaf REL under moderate paucity of water which was not further aggravated with increasing intensity of drought stress (Fig 3B). Drought stress increased reactive oxygen species (ROS) and MDA content in leaves of both sugarcane varieties. In relation to the control, severe water deficit conspicuously increased the MDA content in both genotypes. However, no significant difference was noted between control and moderate stress treatment (Fig 3C). Both varieties showed a clear upsurge in super oxide contents in response to severe water deficit as compared with their respective control treatments. At moderate stress level, the increase in super oxide content was non-significant, with respect to control (Fig 3D). Yuetang 93–159 depicted a considerable increase in hydrogen peroxide content at severe water deficit, with a non-significant response to moderate stress, in relation to control treatment. Conversely, Yunzhe 05–51 depicted a statistically non-significant rise in hydrogen peroxide content at both stress levels (Fig 3E).

### Antioxidant enzyme activities

**Fig 4:**
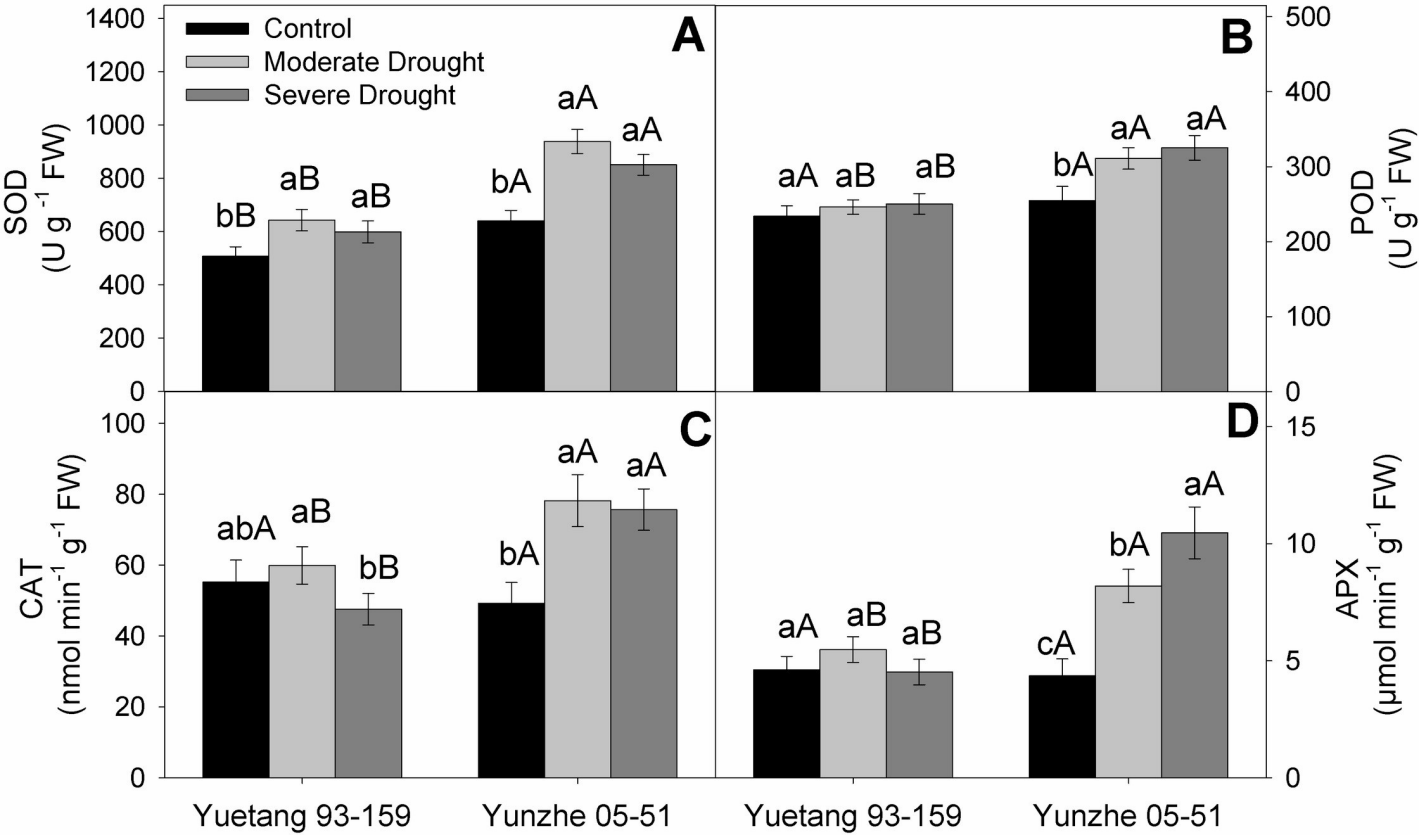
Antioxidant enzyme activities of two sugarcane genotypes in response to drought stress. (A) SOD, (B) POD, (C) CAT, (D) APX. Columns represent the mean values of three replicates ± SD. Bars followed by different letters for genotypes within each water condition (uppercase) as water condition within genotypes (lowercase) differ statistically at P≤5%. Control, moderate drought and severe drought represent 70±5%, 50±5% and 30±5% of soil field capacity, respectively.

Moderate moisture scarcity induced a significant enhancement in leaf SOD activity of both genotypes in relation to the well-watered plants. Further, increase in the intensity of stress slightly declined SOD activities in both genotypes. But the difference between moderate and severe stress treatment was statistically non-significant. Overall, the extent of stress-induced increase in SOD activity was much greater in tolerant genotype (Yunzhe 05–51) than in susceptible variety (Yuetang 93–159) (Fig 4A). As compared with the control, drought stress did not cause any considerable variation in POD activity in leaves of Yuetang 93–159. Whereas Yunzhe 05–51 depicted a considerable rise in POD activity in response to drought stress in contrast with the control treatment. Intensification of stress further enhanced the activity of POD, but the difference between moderate and severe stress treatment was statistically non-significant (Fig 4B). Yuetang 93–159 illustrated a mild increase in CAT activity at moderate water deficit, followed by a considerable decline at severe stress level, in relation to the control treatment. However, the leaves of Yunzhe 05–51 exhibited an obvious surge in CAT activity at both stress level, in comparison with the normally grown plants. Nonetheless, the difference between moderate and severe stress condition was statistically negligible (Fig 4C). APX activity was not affected by moisture deficit treatment in the leaves of Yuetang 93–159. But Yunzhe 05–51 revealed a clear increase in leaf APX activity under moderate moisture deficiency. At increased stress level, the APX activity of Yunzhe 05–51 was further enhanced in contrast to other treatments (Fig 4D).

### Soluble sugars, soluble proteins and proline

**Fig 5:**
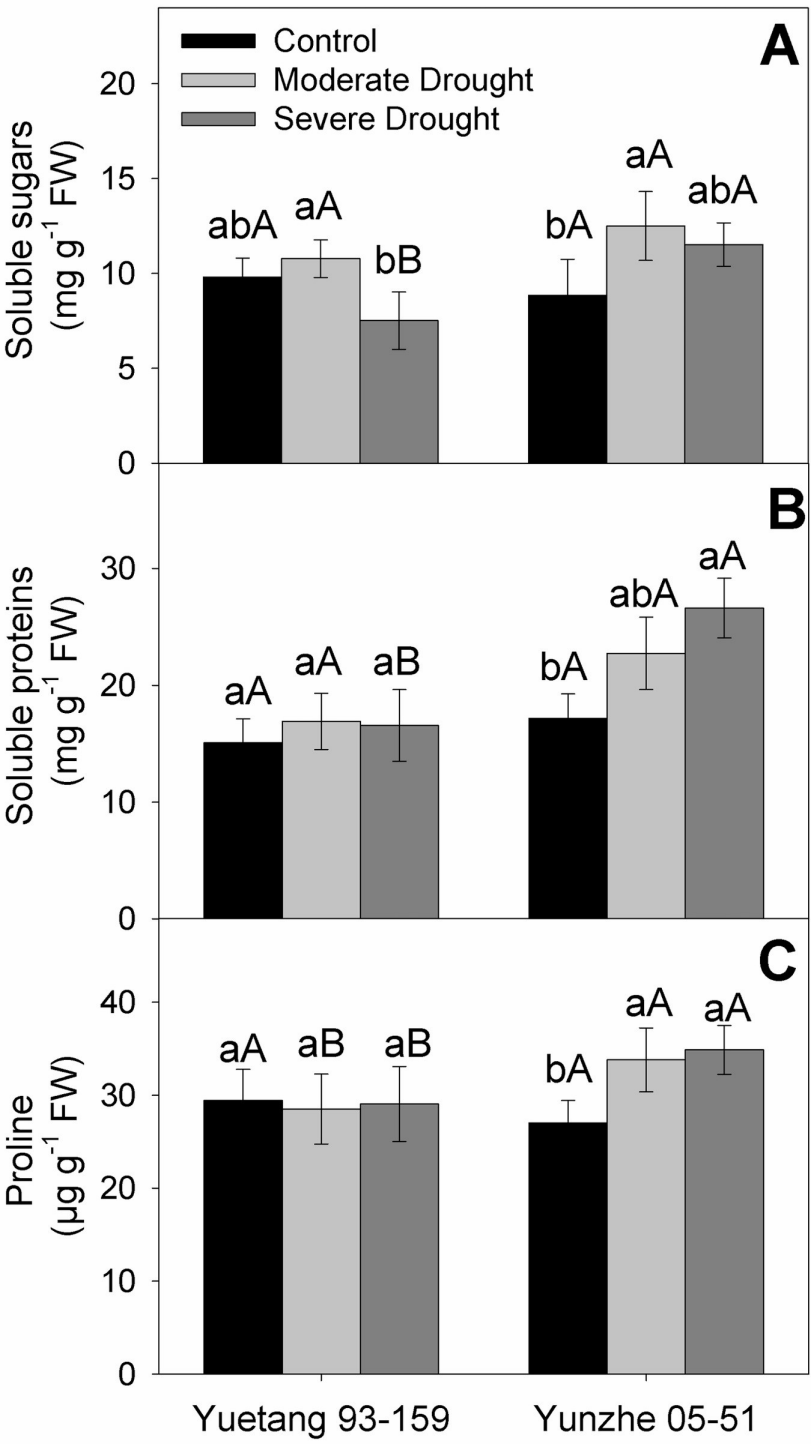
Metabolites of two sugarcane genotypes in response to drought stress. (A) Soluble sugars, (B) Soluble proteins and (C) Proline contents. Columns represent the mean values of three replicates ± SD. Bars followed by different letters for genotypes within each water condition (uppercase) as water condition within genotypes (lowercase) differ statistically at P≤5%. Control, moderate drought and severe drought represent 70±5%, 50±5% and 30±5% of soil field capacity, respectively.

Sensitive genotype (Yuetang 93–159) revealed a statistically negligible increase in soluble sugar contents at moderate stress level in relation to the control treatment, with a considerable subsequent decline at severe water deficit. Contrary to that Yunzhe 05–51 exhibited a significant enhancement in soluble sugar contents at moderate stress treatment. Nonetheless, further increase in stress level did not improve soluble sugar contents of Yunzhe 05–51 (Fig 5A). Moisture deficit induced a very slight and statistically negligible change in soluble protein contents of Yuetang 93–159 with respect to control treatment. Conversely, imposition of drought stress levels progressively improved the soluble protein contents of Yunzhe 05–51 in comparison with the respective control treatment (Fig 5B). For proline content, no statistical difference was observed between stress treatments in Yuetang 93–159. However, an obvious rise in proline content was noted in moisture deficient plants of Yunzhe 05–51 at both stress levels, in comparison with the control treatment. But no statistical variation was detected between moderate and severe moisture deficit in Yunzhe 05-51(Fig 5C).

### Chloroplast ultrastructure

**Fig 6:**
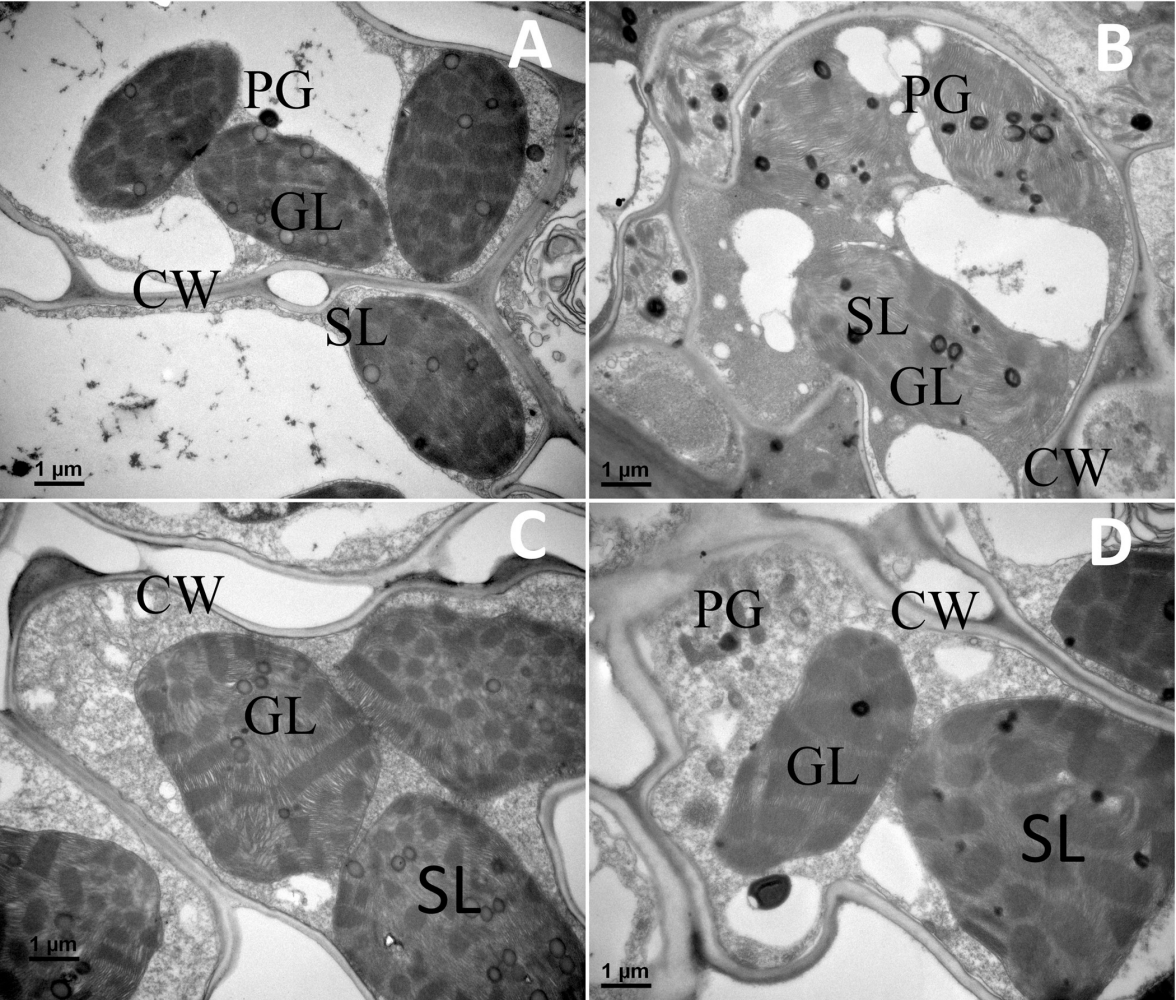
Transmission electron micrographs of chloroplasts of two sugarcane genotypes in response to drought stress. (A) Yuetang 93–159 (Control), (B) Yuetang 93–159 (Severe drought), (C) Yunzhe 05–51 (Control), and (D) Yunzhe 05–51 (Severe drought). Control and severe drought correspond to 70±5% and 30±5% of soil field capacity. CW, cell wall; GL, granum lamellae; SL, stroma lamellae; PG, plastoglobuli.

Transmission electron microscopic images of control mesophyll cells of Yuetang 93–159 and Yunzhe 05–51 genotypes exhibit typically normal structure of chloroplast having several compact grana stackings, well-organized thylakoids, clear thylakoid membranes, and a few plastoglobuli with well composed cell wall (Fig 6A and 6C). Electron microscopic image under severe drought stress (70±5% FC) showed that the amounts of grana and thylakoid membranes were obviously lower, with a greater number of plastoglobuli, than under the control treatment. Under drought stress, the thylakoids are disintegrated with loosely arranged and less compact grana stackings, blurred thylakoid membranes and more plastoglobuli (Fig 6B and 6D). However, it is evident from the ultrastructural view that the chloroplast of the tolerant genotype (Yunzhe 05–51) represents overall better arrangement than that of the sensitive one (Yuetang 93–159). The drought stress proved to be more destructive to the sensitive than to the tolerant genotype.

## Discussion

In the present study, morpho-physiological, biochemical and ultrastructural attributes of two sugarcane genotypes, varying in drought stress tolerance, were examined to understand their response to moisture deficit at stem elongation stage. In the present investigation, plant growth such as height, number of leaves, leaf area as well as fresh and dry biomass of shoot per plant decreased under drought stress, with a more detrimental impact on Yuetang 93–159 (drought sensitive) than on Yunzhe 05–51 (drought tolerant) genotype (Table 1). The Yunzhe 05–51 genotype maintained significantly higher shoot biomass under both water deficit conditions, thereby exhibiting drought hardiness. According to Endres *et al*. [39], considerable genotypic differences were observed for sugarcane plants facing deficit irrigation. Santos *et al*. [40] also revealed obvious varietal differences among sugarcane genotypes for biomass production under reduced moisture supply. Similarly, it has been reported that drought susceptible sugarcane varieties experienced considerable decline in biomass under moisture deficiency [41]. The higher biomass of Yunzhe 05–51 may be attributed to comparatively better CO_2_ assimilation and effective protection from reactive oxygen species (ROS) under stress.

Chlorophyll content is an important indicator for photosynthetic performance of plants [42]. The current study revealed a significant impairment in gas exchange attributes, SPAD and WUE in both sugarcane varieties (Fig 2). However, Yunzhe 05–51 showed higher SPAD value under severe drought stress treatment, which is in conformity with the findings of Augustine *et al*. [43] for transgenic sugarcane plants under drought stress. The stability of chlorophyll contents ensures the functionality of chloroplast and helps the plants maintain photosynthetic activity under stressful environments. Stomatal closure is among the primary responses of plants to water deficit to reduce moisture escape through transpiration, with consequent reduction in growth [44]. Similar observations were recorded for both sugarcane genotypes in the current investigation. It has been demonstrated previously that photosynthetic activity is hampered by both stomatal (limited CO_2_ availability), and non-stomatal factors (oxidative damage to chloroplast) under water deficit [45]. Nonetheless, in the current study, non-significant difference between stomatal conductance of sensitive (Yuetang 93–159) and tolerant (Yunzhe 05–51) genotype under drought stress (Fig 2B) implies that the reduction in photosynthetic performance of sensitive genotype is primarily due to non-stomatal factors (oxidative damage to chloroplast). In the present study, the decrease in *C*i at moderate stress level is due to reduced stomatal conductance in both genotypes, which consequently decreased *P*n. However, at severe drought stress, the increased *C*i may be ascribed to the under-utilization of CO_2_ due to oxidative damage to the photosynthetic apparatus. However, the varieties did not differ significantly for *Ci* which is also evident from the non-significant difference in stomatal conductance of both genotypes. Yuetang 93–159 showed comparatively higher *C*i at severe stress treatment which indicates inefficient CO_2_ assimilation. The higher *C*i indicates a limited CO_2_ utilization, rather than its higher influx. Conversely, non-significant difference in *Ci* between moderate and severe stress level in Yunzhe 05–51 implies that this genotype is better capable of maintaining photosynthetic activity, with enhanced WUE, even at severe moisture deficit (Fig 2). The severe impairment in photosynthetic activity and WUE of Yuetang 93–159 might be ascribed to the disruption of Calvin cycle in the photosynthetic electron transport chain [46] due to over production of ROS, coupled with inefficient ROS scavenging system [18]. Contrarily, better photosynthetic performance of Yunzhe 05–51 might be due to efficient antioxidant defense mechanism (Fig 4). Decrease in amount and activity of Rubisco might be a possible explanation for drought-induced decline in photosynthesis at severe stress level [47]. Generally, it is deduced that Yunzhe 05–51 possesses more efficient protective mechanism against drought stress than does Yuetang 93–159. The results of the current probe indicate that comparatively better tolerance of Yunzhe 05–51 to water deficit is mainly linked with the non-stomatal factors which is evident from the non-significant difference in stomatal conductance and significant difference in antioxidant enzyme activities of both genotypes. Similar findings were reported by Reis *et al*. [48] while comparing the photosynthetic performance of drought tolerant and sensitive plants of sugarcane. Osmoregulation is an important mechanism to maintain cell turgidity in plants against moisture depletion [49]. The plants of both genotypes considerably decreased relative water content (RWC) at severe stress level in relation to their respective control treatment (Fig 3A). RWC is an important measure to evaluate tolerance of plants to drought stress. Previous studies illustrated that moisture deficit significantly reduced leaf RWC in sugarcane [21, 50, 51], wheat [52] and beans [53]. Previously, Cia *et al*. [50] also reported significant differences in leaf RWC in sugarcane under water scarcity.

Drought-induced oxidative damage is a well-established fact in plants and its intensity demonstrates the degree of stress tolerance [54]. In current study, increased MDA, O^-^_2_, and H_2_O_2_ contents, accompanied by elevated REL, were observed in both genotypes under water deficit stress (Fig 3). The drought-induced generation of ROS has previously been reported in tomato [55], common beans [53] and sugarcane [21], which was correlated with the deterioration of membrane system and its magnitude was used as a stress tolerance index. Gill *et al*. [34] narrated that over-generation of ROS is detrimental to plant cells that ends up in elevated lipid peroxidation, relative electrolyte leakage (REL) and DNA damage. ROS may also serve as signaling molecules in stress responses, but their higher concentration is toxic to plant cells [56]. The most commonly observed outcomes of ROS include loss of pigments, reduced gaseous exchange and deterioration of proteins and RNA [57].

The role of the antioxidants is crucial to protect the cells from ROS damage in case of disrupted redox balance under stress. SOD plays a pivotal role to protect the plant cells against superoxide (O^-^_2_) radicals [58]. The current study reveals comparatively higher SOD activity in the leaves of tolerant genotype (Yunzhe 05–51) at all stress levels, indicating better ROS scavenging ability than the sensitive variety. The enhanced ROS scavenging ability of tolerant sugarcane plants inhibits oxidative damage and safeguards membrane system by curbing lipid peroxidation under stressful conditions [59]. To cope with the higher accumulation of superoxide, SOD serves as a sink for excess electrons generated in response to stress. In another varietal trial, drought tolerance of maize genotypes was attributed to the elevated activity of SOD [60]. Jain *et al*. [61] observed a higher expression of SOD gene under drought stress in sugarcane. Previous studies have also revealed the similar observations and reported that better performance of drought tolerant sugarcane genotypes might be attributed to the improved SOD activity [21]. CAT and other peroxidases reduce H_2_O_2_ to H_2_O. In current study, both sugarcane genotypes depicted rise in CAT activity in response to moderate drought conditions (Fig 3C). However, this surge in CAT activity could combat excess H_2_O_2_ generation only in Yunzhe 05–51, as indicated by non-significant increase in H_2_O_2_ content under moderate and severe stress, in relation to control. The unchanged H_2_O_2_ level in tolerant cultivar under stress, suggests that CAT and other peroxidases (POD, APX) efficiently detoxified H_2_O_2_, produced in excess (Fig 3E). This hypothesis is also supported by the elevated activity of CAT, POD and APX in Yunzhe 05–51 at increased stress levels (Fig 4). Further, reduced CAT and unchanged POD and APX activities in Yuetang 93–159 leaves indicate its incapability to combat drought stress, which is also corroborated with increased ROS and MDA contents at severe stress level. The results clearly indicated that the antioxidant defense system of Yuetang 93–159 does not have the potential to cope with the over-generation of ROS and MDA at higher stress level. Jain *et al*. [61] also observed the enhanced activities of antioxidants in sugarcane, in response to moisture deficient growth environment. In another study, better performance of tolerant rice cultivar was attributed to the improved activity of antioxidant enzymes to ameliorate the drought stress [62]. Similar outcomes of drought stress were observed in different wheat genotypes [63]. The enhancement in the activities of antioxidant enzymes against ROS suggests that a robust response to water deficit occurred in Yunzhe 05–51 at both stress levels. The main consequences of the stress on both genotypes can be clearly observed in growth traits (Table 1) and photosynthetic performance (Fig 2), whereby it is clear that the Yunzhe 05–51 performed better than Yuetang 93–159 did under both stress levels. The results of the present study indicate that excess ROS, generated under drought stress, were effectively detoxified by the antioxidants defense system of tolerant genotype (Yunzhe 05–51).

The accumulation of organic solutes like soluble sugars and proline in plants is instigated by water deficit to maintain osmotic balance [54]. Apart from their role in osmotic adjustment, compatible solutes are also reported to improve plants’ defense system against ROS-induced oxidative damage [64] which consequently improves the capability of plants to withstand stressed environment [65]. Higher proline concentration in Yunzhe 05–51 seems to have osmo-regulatory effect in stressed plants, which might be an evolutionary development to enable them to adapt to hostile environment, as revealed by previous reports on sugarcane [40, 66, 67] and maize varieties [68, 69] in response to moisture deficit. Drought-induced accumulation of osmo-regulators protects cell structure against desiccation and directly contributes to maintain osmotic balance [70, 71]. After the stress period is over, the accumulated proline may be used as a source of energy for the recovery of plant’s physiological activity [72]. Molinari *et al*. [73] reported that over-expression of P5CS (Pyrroline-5-Carboxylate Synthase), a proline synthesizing enzyme, protected the transgenic plants of sugarcane from drought-induced oxidative damage. Yunzhe 05–51 maintained higher leaf proline concentrations under drought stress (Fig 5C), which indicates that this genotype is better capable of tolerating dehydration, and shows less deterioration in growth attributes than the sensitive variety Yuetang 93–159 (Table 1), in relation with their respective control treatments. Accumulation of proline in plants for adaptation to different environmental stresses is a well-known phenomenon [74]. According to Kishor *et al*. [75], proline protects the plant cells from damaging effects of singlet oxygen and free radicals, which consequently plays a role in stabilizing DNA, proteins, membrane system and sub-cellular structures. Jain *et al*. [61] reported up-regulation of P5CS gene, responsible for proline synthesis, under drought stress in sugarcane. In the present study, Yunzhe 05–51 exhibited higher values of leaf soluble sugar contents in response to moisture deficit. Previous studies on sugarcane have also revealed a rise in total soluble sugars and indicated their effective role in osmotic regulation under dehydration [71, 76, 77]. The key functions of soluble sugars in stress amelioration include osmotic adjustment, storage of carbon and ROS scavenging [78]. Santos *et al*. [40] observed a significant increase in soluble sugar contents of drought tolerant sugarcane genotypes under water scarce conditions, which is in agreement with the current study results. Previous studies also revealed higher soluble sugar accumulation in drought-tolerant barley [54] and cotton [11] genotypes in response to moisture stress. Thus, it is proposed that Yunzhe 05–51 may acquire more osmotic protection than Yuetang 93–159 under drought stress due to higher soluble sugar contents. In the current experiment, tolerant genotype (Yunzhe-05-51) depicted higher leaf soluble protein content than the sensitive one (Yuetang 93–159) under drought stress. Li *et al*. [79] observed a considerable enhancement in soluble protein contents of cotton leaves under drought stress and suggested their important physiological role in stress tolerance during early growth stages. Santos *et al*. [40] also observed a 30% rise in soluble protein contents of drought tolerant sugarcane genotypes, with respect to control treatment, under water deficit conditions. Proteolysis usually serves to provide amino acids to synthesize proteins in response to stress [80]. In the current experiment, the improved protein contents may be an outcome of increased protease activity. The activity of protease may be triggered by the release of proteins because of membrane injury, for their proteolysis. The resultant amino acids may be utilized for enhanced production of antioxidant enzymes, which are also protein in nature. Consequently, the synthesis of antioxidant enzymes and other stress responsive proteins like dehydrins, might be a possible explanation for enhanced leaf protein content in stressed sugarcane plants of tolerant genotype (Yunzhe 05–51) [54].

Visualizing sub-cellular structural changes in the leaves of drought stressed plants is a useful measure to unveil the fundamental mechanism related to drought tolerance. Being main site for ROS generation, chloroplast is highly prone to oxidative deterioration [19]. Swelling of thylakoid and lipid droplets are the generalized symptoms of various stresses in plants [81]. Formation of plastoglobuli occurs because of breaking down of thylakoid membranes [82]. In the current study, damage to chloroplast occurred as a result of oxidative stress caused by drought. Activities of antioxidant enzymes may safeguard the photosynthetic apparatus of plants against oxidative injury under stress [59], which is evident from the ultra-structural view of the chloroplast in tolerant sugarcane genotype. Comparatively better arrangement of chloroplast structure in Yunzhe 05–51 under drought stress may be attributed to the effective antioxidant enzyme protection system against ROS in this genotype. Similar genotypic variations in chloroplast anatomy of sugarcane [3] and cotton [11] under drought stress have been reported previously.

## Conclusion

Results of the current study revealed that moisture deficit significantly reduces growth attributes, photosynthetic performance and chlorophyll contents in leaves of both sugarcane varieties, depicting obvious inhibition in sensitive genotype (Yuetang 93–159). The current study demonstrated that Yunzhe 05–51 is better tolerant to drought stress than Yuetang 93–159, and the tolerance was linked to effective antioxidant enzyme defense system. Further, accumulation of soluble sugars, soluble proteins and proline confers tolerance to drought stress through osmotic regulation to maintain cell turgidity, which might enable the plant to adapt to unfavorable environment. Additionally, ultrastructure of chloroplast of tolerant genotype (Yunzhe 05–51) was less affected by moisture deficit. The present findings may serve as a basis for the development of drought tolerant cultivars. However, further studies are recommended to uncover the underlying molecular mechanism of drought stress tolerance.
